# Chemical Comparison of Monk Fruit Products Processed by Different Drying Methods Using High-Performance Thin-Layer Chromatography Combined With Chemometric Analysis

**DOI:** 10.3389/fnut.2022.887992

**Published:** 2022-05-02

**Authors:** Hui-Jie Hong, Qi Yang, Qiao Liu, Fong Leong, Xiao-Jia Chen

**Affiliations:** ^1^State Key Laboratory of Quality Research in Chinese Medicine, Institute of Chinese Medical Sciences, University of Macau, Macau, Macao SAR, China; ^2^Zhuhai UM Science and Technology Research Institute, Zhuhai, China

**Keywords:** monk fruit, *Siraitia grosvenorii*, high performance thin layer chromatography, drying method, chemical characteristics, chemometric analysis

## Abstract

Monk fruit, also named Luo Han Guo, is the fruit of *Siraitia grosvenorii* (Swingle) C. Jeffrey ex A. M. Lu et Z. Y. Zhang and has been used as both food and traditional Chinese medicine. Due to preservation concerns, monk fruit is usually processed by hot-air drying or using low-temperature techniques after harvest. In this study, high-performance thin-layer chromatography (HPTLC) method was developed for the analysis of 13 mogrosides, 1 flavonoid, and 3 sugars in monk fruit products. Then chemometric analysis was applied to investigate the chemical characteristics in the samples dried by different methods. The results showed that the contents of mogroside V, 11-oxo-mogroside V, isomogroside V, and sucrose in monk fruits dried at low temperature were much higher than those in traditional hot-air drying samples, which was also confirmed by HPTLC-scanning. These findings indicate that HPTLC combined with chemometric analysis provides a reliable tool to understand the chemical differences between the monk fruit products processed by different drying methods, which will be helpful for their quality evaluation.

## Introduction

Monk fruit, also known as Luo Han Guo, is the fruit of *Siraitia grosvenorii* (Swingle) C. Jeffrey ex A. M. Lu et Z. Y. Zhang (1). It is mainly cultivated in Guangxi, China, and has been used as a food ingredient, beverage, and traditional medicine for centuries. Because of its good safety and high sweetness, monk fruit had been approved as a food sweetener by China Food and Drug Administration and awarded the “generally regarded as safe” (GRAS) status by the U.S. Food and Drug Administration (2). It is used as a sugar-free food additive in low-calorie health-promoting drinks, and also as a substitute for sweeteners in health foods for patients with obesity and diabetes (2). As a traditional Chinese medicine, monk fruit has been used for the treatment of dry cough, sore throat, and constipation (1). Recent pharmacological studies have also shown that monk fruit exhibits anti-diabetic (3–5), anti-cancer (6, 7), anti-inflammatory (8–10), and neuroprotective effects (11–13).

The bioactive and nutritional ingredients in monk fruit include triterpene glycosides, flavonoids, carbohydrates, proteins, fats, vitamins, and minerals. Mogrosides are a group of cucurbitane-type triterpene glycosides that are the major bioactive compounds in monk fruit. The mixture of mogrosides is 300 times sweeter than sucrose (14), but only mogrosides with mogrol aglycone and with more than three sugar moieties possess the sweet taste (15, 16). Flavonoids are also important compounds in monk fruit and exert antibacterial and antioxidant effects (17). In addition, there are various sugars found in monk fruit. In different varieties of monk fruits, the total sugar content accounted for 25–38% of dry weight. Among them, the contents of fructose and glucose were 10–17% and 5–15%, respectively (18).

**FIGURE 1:**
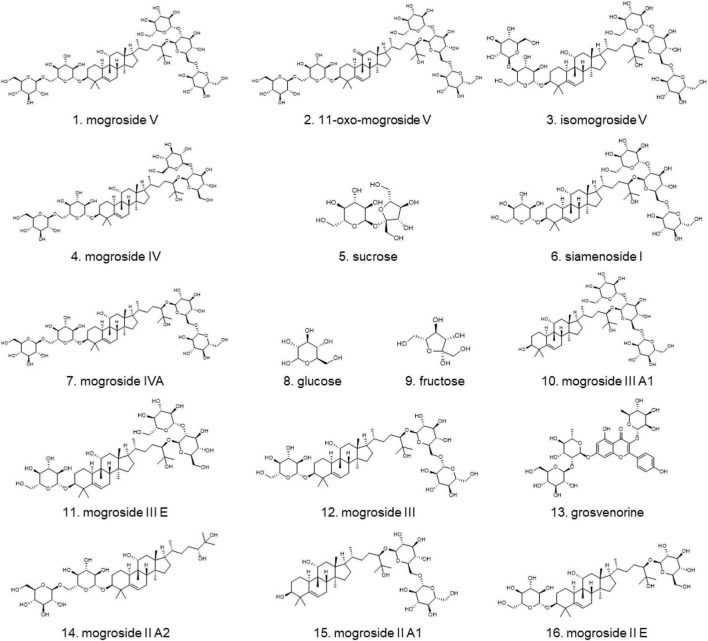
Chemical structures of 16 investigated compounds.

Monk fruit usually needs a drying process before further use to inhibit microbial growth and extend the shelf life. Traditionally, monk fruit is dried by hot air at 45–70^°^C for 6–8 days to remove the moisture, after which the outer surface of the monk fruit will turn to dark yellow or brown (Supplementary Figure 1), and the taste may be slightly bitter. To obtain better appearance, taste, and quality, low temperature techniques, such as freeze-drying, freeze-vacuum drying, microwave drying, microwave-vacuum drying, microwave-vacuum infrared drying, and freezing followed by microwave-vacuum drying are used for drying monk fruit (19). Currently, both types of monk fruit products are widely available in the market. It is reported that drying methods may greatly affect the bioactive and nutritional components of monk fruit. Lu et al. found that monk fruits dried with freezing contained higher content of mogroside V than those dried under high temperatures (20). In addition, the contents of 10 mogrosides in monk fruits processed by vacuum drying method were markedly higher than those in traditional drying samples (21). Wang et al. indicated that high-temperature drying treatment resulted in a significant decrease in sucrose and glucose concentrations compared with freeze-dried fruit (22). However, these reports employed high-performance liquid chromatography which mainly focused on only mogrosides or sugars due to their different polarity. Simultaneous analysis of multiple components may reflect the quality more comprehensively. Therefore, in this study, 16 compounds in monk fruit products, such as 13 mogrosides [mogroside V (**1**), 11-oxo-mogroside V (**2**), isomogroside V (**3**), mogroside IV (**4**), siamenoside I (**6**), mogroside IV A (**7**), mogroside III A1 (**10**), mogroside III E (**11**), mogroside III (**12**), mogroside II A2 (**14**), mogroside II A1 (**15**) and mogroside II E (**16**)], 1 flavonoid [grosvenorine (**13**)], and 3 sugars [sucrose (**5**), glucose (**8**), and fructose (**9**)] (Figure 1) were analyzed by high-performance thin-layer chromatography (HPTLC), which has the advantages of high selectivity for complex components and high efficiency for comparing a large number of samples simultaneously. Then, chemometric analysis was performed to compare the chemical differences in monk fruit products processed by different drying methods.

## Materials and Methods

### Materials and Chemicals

Monk fruit samples of different sizes (S: small; M: medium; L: large; and XL: extra-large) and processed by different drying methods (HT: high temperature and LT: low temperature) were collected at pharmacies from different locations in China. All monk fruit samples were produced in Guilin, Guangxi, and their information is listed in Supplementary Table 1. The botanical origin of materials was identified by Dr. Xiao-Jia Chen, one of the authors. All voucher specimens were deposited at the Institute of Chinese Medical Sciences, University of Macau, Macao SAR, China.

All chemicals and solvents were of analytical grade. Ethanol and acetic acid were bought from Xilong Scientific Co., Ltd. (Shantou, China), and sulfuric acid (98%) was acquired from Merck (Darmstadt, Germany). Ethyl acetate was purchased from ACI Lascan Limited (Bangkok, Thailand). Methanol was obtained from Damao Chemical Reagent Factory (Tianjin, China) while *n*-butanol was purchased from Tianjin Fuyu Chemical Reagent Factory (Tianjin, China). All mogrosides (mogroside V, 11-oxo-mogroside V, isomogroside V, mogroside IV, siamenoside I, mogroside IV A, mogroside II A1, mogroside II E, mogroside II A2, mogroside III, mogroside III A1, and mogroside III E) and grosvenorine were purchased from Chengdu Purify Co., Ltd. (Chengdu, China). Glucose, sucrose, and fructose were acquired from Chengdu Pufei De Biotech Co., Ltd. (Chengdu, China). All aqueous solutions were prepared with deionized water purified by the Millipore Milli Q-Plus system (Millipore, Billerica, MA, United States).

### Sample Preparation

Dried powdered samples (2.0 g) were sonicated with 40 ml of mill-Q water for 30 min, then the extract was centrifugated at 3,000 rpm for 5 min. The supernatant was collected and extracted with 20 ml of water-saturated *n*-butanol two times. Then, the *n*-butanol fractions were combined and evaporated to dryness in a rotary evaporator. The residue was dissolved in 2.0 ml of methanol and filtered through a 0.22 μm nylon membrane for further experiment.

### Standard Solutions Preparation

Separate stock solutions (1 mg/ml) of the 16 compounds were prepared in methanol or water. Then, two mixed standard solutions were prepared, respectively, by mixing equal volumes of the corresponding stock solutions. Mixed standard solution 1 (MS1) was composed of mogroside V, isomogroside V, mogroside IV, siamenoside I, mogroside IV A, mogroside II A1, mogroside II E, mogroside II A2, mogroside III, mogroside III A1, mogroside III E, and glucose at the final concentration of 0.08 mg/ml, while the other components (11-oxo-mogroside V, grosvenorine, fructose and sucrose) were mixed to form the mixed standard solution 2 (MS2) at the final concentration of 0.25 mg/ml.

### High-Performance Thin-Layer Chromatography Analysis

A CAMAG TLC system (CAMAG, Switzerland) containing an automatic thin-layer chromatography (TLC) sampler 4 with a 25 μl syringe, an automatic developing chamber, a chromatogram immersion device III, a TLC plate heater III, a TLC visualizer equipped with visionCATS (version 2.5) software, and a TLC scanner 4 was employed for the analyses. To maintain a similar application amount of each standard on the plate, different application volumes were used. MS 1 (22 μl), MS 2 (8 μl), and 28 sample solutions (2 μl) were applied as 8 mm bands and 8 mm from the bottom edge on HPTLC silica gel 60 F_254_ plates (20 cm × 10 cm, Merck, Darmstadt, Germany). After sample application, the plate was pre-saturated with the mobile phase of *n*-butanol - water-ethanol-acetic acid (7:1:1:0.2, v/v/v/v) for 30 min in a glass double-twin trough chamber, then the plate was developed with the same developing agent to 80 mm from the bottom edge. After drying, the plate was then immersed in 10% sulfuric acid in ethanol solution for 1 s and heated at 105^°^C for 10 min on a TLC plate heater. All plate images were documented under white light and UV 366 nm. Then, the plate was scanned at 290 nm with a scanning speed of 20 mm/s and a slit dimension of 5 × 0.2 mm being employed.

### Data Analysis

The obtained HPTLC images were uploaded to the rTLC V.1.0 program^1^ for processing (23). The data of every track in the HPTLC images under UV 366 nm were extracted by adjusting the parameters based on sample application. Then, the data matrix of the red channel consisting of sample code, variables ID (R_f_ region), and pixel intensity was exported as csv. format and further analyzed using SIMCA software (version 14.1, Umetrics).

## Results and Discussion

### Optimization of the High-Performance Thin-Layer Chromatography Conditions

**FIGURE 2:**
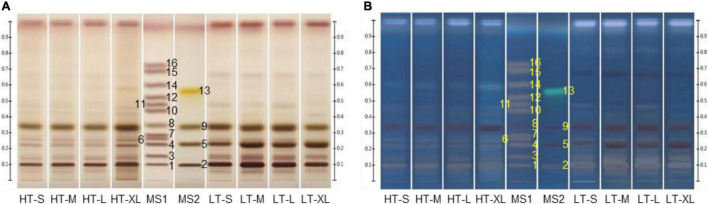
Representative high-performance thin-layer chromatography (HPTLC) chromatograms of mixed standards and monk fruit samples. Plates were immersed into 10% sulfuric acid in ethanol solution for derivatization and photographed under **(A)** white light and **(B)** UV 366 nm. HT, monk fruit dried at high temperature; LT, monk fruit dried at low temperature; S, small; M, medium; L, large; XL, extra-large; MS1 and MS2, mixed standards. (1) Mogroside V (R_f_ 0.10), (2) 11-oxo-mogroside V (R_f_ 0.10), (3) isomogroside V (R_f_ 0.15), (4) mogroside IV (R_f_ 0.23), (5) sucrose (R_f_ 0.23), (6) siamenoside I (R_f_ 0.26), (7) mogroside IV A (R_f_ 0.29), (8) glucose (R_f_ 0.35), (9) fructose (R_f_ 0.34), (10) mogroside III A1 (R_f_ 0.44), (11) mogroside III E (R_f_ 0.48), (12) mogroside III (R_f_ 0.53), (13) grosvenorine (R_f_ 0.56), (14) mogroside II A2 (R_f_ 0.60), (15) mogroside II A1 (R_f_ 0.69), and (16) mogroside II E (R_f_ 0.73).

Different developing agents were optimized to achieve good separation. The mobile phase in the Chinese Pharmacopoeia, *n*-butanol-ethanol-water (8:2:3, v/v/v) (1) was first tried, but the mogrosides with the same number of sugar units were hard to be separated. By using the upper layer of *n*-butanol–ethyl acetate-water (4:2:4, v/v/v), the separation of similar mogrosides was greatly improved but the R_f_ value of mogroside V was too low. With *n*-butanol-water ethanol (7:1:1, v/v/v), the R_f_ value of mogroside V increased but the chromatographic trailing of mogroside V in samples existed. Finally, a satisfactory result was presented by using *n*-butanol-water-ethanol-acetic acid (7:1:1:0.2, v/v/v/v) as the mobile phase. However, under this condition, mogroside V and 11-oxo-mogroside V, mogroside IV, and sucrose, as well as fructose and glucose were still hard to be separated (Figure 2). Therefore, unseparated reference standards were prepared in two different solutions to avoid the overlapping of structural analogs.

### Comparison of Monk Fruit Samples Dried at High Temperature and Low Temperature by High-Performance Thin-Layer Chromatography Images Directly

Monk fruit samples of different sizes and dried at different temperatures were analyzed by the developed HPTLC method. As shown in Figure 2 and Supplementary Figure 2, there was no significant difference among the samples of different sizes processed by the same drying method, but the drying method did have influence on the chemical compositions of monk fruit. Mogroside V and 11-oxo-mogroside V, isomogroside V, mogroside IV, siamenoside I, glucose, and fructose were observed in all samples, while sucrose was only detected in LT groups. The major differences between HT and LT samples were in the range of R_f_ 0.10–0.25, in which the contents of mogroside V, 11-oxo-mogroside V, and isomogroside V in LT groups were much higher than those in HT samples. Moreover, mogroside IV A could be found in HT samples but was hardly detected in most of the LT samples. These results were consistent with the previous reports (20–22).

### Comparison of Monk Fruit Samples Dried at High Temperature and Low Temperature by Chemometric Approaches

**FIGURE 3:**
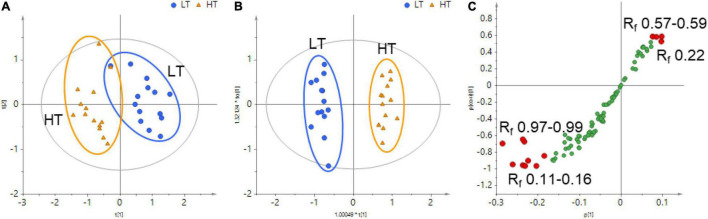
**(A)** Principal component analysis (PCA) score plot, **(B)** orthogonal partial least square-discriminant analysis (OPLS–DA) score plot, and **(C)** OPLS–DA S-plot of HT and LT samples based on the data extracted from the whole HPTLC chromatograms. The variables contributing most to the differences were highlighted with red-filled circles.

Although an obvious difference in the HPTLC profiles could be visually inspected between HT and LT monk fruit samples, visual observation was insufficient to discriminate between these two groups. Therefore, chemometric approaches were applied to further explore the chemical characteristics of the two types of monk fruits. The whole HPTLC chromatograms under 366 nm were processed by rTLC program to generate a dataset involving sample code, R_f_ region, and pixel intensity, and a total of 91 variables were extracted across samples (Supplementary Table 2). Principal component analysis (PCA) was first applied to obtain a basic insight into the specific grouping patterns between the monk fruit samples. As shown in Figure 3A, the PCA score plot showed a tendency to separate the monk fruit samples in terms of the drying method. The model is composed of five principal components with *R^2^X* value of 0.820 and *Q*^2^ value of 0.646, indicating good fitness and prediction of the constructed PCA model.

To further characterize the differences in monk fruit samples treated with different drying methods, orthogonal partial least square-discriminant analysis (OPLS-DA) was subsequently conducted to sharpen the separation between the groups in PCA. As shown in Figure 3B, 28 monk fruit samples were divided into two classes based on different drying methods. All the samples fell within the Hotelling T2 (0.95) ellipse, where the model fit parameters were 0.699 of *R*^2^*X*, 0.974 of *R*^2^*Y*, and 0.929 of *Q*^2^, indicating that the OPLS-DA model established in this study has high goodness-of-fit and predictive ability.

An S-plot was constructed following the OPLS-DA to reveal the chemical components contributing mostly to the differences between the HT and LT groups. In this plot, the data points at the two ends of the S-shaped curve made the greatest contribution to the two-group separation with the highest confidence, which were highlighted in red in Figure 3C. The bands of R_f_ 0.11–0.16 and R_f_ 0.97–0.99 at the bottom-left corner and the bands of R_f_ 0.57–0.59 and R_f_ 0.22 at the top-right corner of the S-shaped curve were considered as the characteristic components contributing most to the distinction of HT and LT groups. Compared with the reference standards, the bands of R_f_ 0.10, 0.15, and 0.22 contained mogroside V and 11-oxo-mogroside V, isomogroside V, and mogroside IV and sucrose are not separated by the HPTLC method and they correspond to the band of Rf 0.22, respectively.

PCA and OPLS-DA based on the HPTLC data of the 16 compounds with reference standards were also conducted. As shown in Supplementary Figure 3, the two types of monk fruit samples were also clearly separated, and mogroside V, 11-oxo-mogroside V, and isomogroside V were the most discriminating between the two groups. Compared with the analysis based on the 16 compounds, the results based on the data from the whole chromatograms could reflect the differences between groups more comprehensively, and more discriminating variables could be found. However, it was difficult to identify the unknown bands under the current HPTLC condition due to the low contents or the poor separation. Further isolation and identification by other chromatographic and detection techniques should be performed in the future.

### Semi-Quantification by High-Performance Thin-Layer Chromatography-Scanning

**FIGURE 4:**
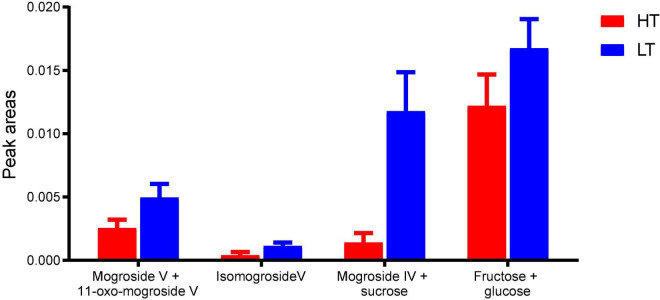
Peak areas of selected mogrosides and sugars in HT and LT samples by HPTLC-scanning.

Based on the above results, mogroside V, 11-oxo-mogroside V, isomogroside V, mogroside IV and sucrose were found to be the characteristic compounds to distinguish the two types of monk fruits. Furthermore, fructose and glucose were also important nutritional ingredients in monk fruit. Therefore, these compounds were semi-quantified by HPTLC-scanning to verify their differences in HT and LT monk fruit samples. Due to the poor resolution, the bands were scanned in four groups, i.e., mogroside V and 11-oxo-mogroside V; isomogroside V; mogroside IV and sucrose were scanned as one group; as well as fructose and glucose. As shown in Figure 4, the peak areas of all the investigated compounds in LT samples were significantly higher than those in HT samples, which further confirmed the influence of different drying methods on the chemical components of monk fruit samples.

### Possible Factors Affecting the Chemical Compositions of the Processed Monk Fruit

During the drying process, many factors, such as temperature, enzymes, and intermediate products may affect the chemical compositions of the processed monk fruit. It was reported that several enzymes, such as squalene epoxidase, triterpenoid synthases, epoxide hydrolases, cytochrome P450s, and UDP-glucosyltransferases, were involved in the biosynthesis of mogrosides, such as mogroside V, 11-oxo-mogroside V, and isomogroside V (24, 25). These enzymes may be inhibited at high temperature, which may decrease the content of these mogrosides in HT samples (26). While for the sugars, on the one hand, high temperature may lead to the degradation of sucrose, oligosaccharides, and polysaccharides, resulting in a decrease of sucrose levels and an increase in glucose and fructose contents. On the other hand, the Maillard reaction, a type of non-enzymatic browning, may take place between the reducing sugars and amino acids that are rich in monk fruit. The reaction may proceed more rapidly at high temperatures, thus decreasing the levels of glucose and fructose in HT samples (27). Under the influences of these effects, the contents of the glucose and fructose in HT samples were lower than those in LT samples. However, how the drying methods influence the chemical components of monk fruit is still not clear, which needs further exploration in the future.

## Conclusion

In this study, HPTLC combined with chemometric approaches were used to compare the chemical components of monk fruit products dried at high and low temperatures, respectively. As a result, the contents of mogroside V, 11-oxo-mogroside V, isomogroside V, mogroside IV and sucrose in monk fruits dried at low temperature were higher than those in traditional hot-air drying samples. In the future, laboratory simulated studies should be conducted to explore the relationships between the drying methods and the chemical compositions of monk fruit. In addition, the bioactivities of monk fruit processed by different drying methods should be further studied as well.

## Data Availability Statement

The original contributions presented in the study are included in the article/Supplementary Material, further inquiries can be directed to the corresponding author/s.

## Author Contributions

H-JH and QY performed the experiment and prepared the manuscript. QL and FL analyzed the data. X-JC designed the study and finalized the manuscript. All authors read and approved the final manuscript.

## Conflict of Interest

The authors declare that the research was conducted in the absence of any commercial or financial relationships that could be construed as a potential conflict of interest.

## Publisher’s Note

All claims expressed in this article are solely those of the authors and do not necessarily represent those of their affiliated organizations, or those of the publisher, the editors and the reviewers. Any product that may be evaluated in this article, or claim that may be made by its manufacturer, is not guaranteed or endorsed by the publisher.

## Footnotes

1
http://shinyapps.ernaehrung.uni-giessen.de/rtlc/

## References

[1] Chinese Pharmacopoeia Commission. Siraitiae Fructus. In: *Chinese Pharmacopoeia Commission editor. Chinese Pharmacopoeia of the People’s Republic of China.* Vol. 1. Beijing: China Medical Science and Technology Press (2020). p. 221–2.

[2] Gong X Chen NMH Ren K Jia JY Wei KH Zhang L The fruits of *Siraitia grosvenorii*: a review of a chinese food-medicine. *Front Pharmacol.* (2019) 10:1400. 10.3389/fphar.2019.01400 31849659 PMC6903776

[3] Liu HS Wang CC Qi XY Zou J Sun ZD. Antiglycation and antioxidant activities of mogroside extract from *Siraitia grosvenorii* (Swingle) fruits. *J Food Sci Technol.* (2018) 55:1880–8. 10.1007/s13197-018-3105-2 29666541 PMC5897311

[4] Zhang YL Zhou GS Peng Y Wang MY Li XB. Anti-Hyperglycemic and anti-hyperlipidemic effects of a special fraction of Luohanguo extract on obese T2DM rats. *J Ethnopharmacol.* (2020) 247:112273. 10.1016/j.jep.2019.112273 31586692

[5] Liu HS Qi XY Yu KK Lu AJ Lin KF Zhu JJ AMPK activation is involved in hypoglycemic and hypolipidemic activities of mogroside-rich extract from *Siraitia grosvenorii* (Swingle) fruits on high-fat diet/streptozotocin-induced diabetic mice. *Food Funct.* (2019) 10:151–62. 10.1039/c8fo01486h 30516208

[6] Liu C Dai LH Dou DQ Ma LQ Sun YX. A natural food sweetener with anti-pancreatic cancer properties. *Oncogenesis.* (2016) 5:e217. 10.1038/oncsis.2016.28 27065453 PMC4848839

[7] Liu C Dai LH Liu YP Rong L Dou DQ Sun YX Antiproliferative activity of triterpene glycoside nutrient from monk fruit in colorectal cancer and throat cancer. *Nutrients.* (2016) 8:360. 10.3390/nu8060360 27304964 PMC4924201

[8] Shen JK Shen D Tang Q Li ZJ Jin XM Li CM. Mogroside V exerts anti-inflammatory effects on fine particulate matter-induced inflammation in porcine alveolar macrophages. *Toxicol Vitro.* (2022) 80:105326. 10.1016/j.tiv.2022.105326 35134483

[9] Liu YS Wang J Guan X Yu D Huangfu MJ Dou T Mogroside V reduce OVA-induced pulmonary inflammation based on lung and serum metabolomics. *Phytomedicine.* (2021) 91:153682. 10.1016/j.phymed.2021.153682 34483017

[10] Sung YY Kim SH Yuk HJ Yang WK Lee YM Son E *Siraitia grosvenorii* residual extract attenuates ovalbumin-induced lung inflammation by down-regulating IL-4, IL-5, IL-13, IL-17, and MUC5AC expression in mice. *Phytomedicine.* (2019) 61:152835. 10.1016/j.phymed.2019.152835 31035047

[11] Luo HJ Peng CX Xu XF Peng YT Shi F Li QH The protective effects of mogroside V against neuronal damages by attenuating mitochondrial dysfunction via upregulating Sirtuin3. *Mol Neurobiol.* (2022): [Online ahead of print] 10.1007/s12035-021-02689-z 35040040

[12] Wang H Meng GL Zhang CT Wang H Hu M Long Y Mogrol attenuates lipopolysaccharide (LPS)-induced memory impairment and neuroinflammatory responses in mice. *J Asian Nat Prod Res.* (2020) 22:864–78. 10.1080/10286020.2019.1642878 31347387

[13] Chen GL Liu CH Meng GL Zhang CT Chen F Tang SS Neuroprotective effect of mogrol against Aβ_1–42_ induced memory impairment neuroinflammation and apoptosis in mice. *J Pharm Pharmacol.* (2019) 71:869–77. 10.1111/jphp.13056 30585314

[14] Fang CN Wang QQ Liu XY Xu GW. Metabolic profiling analysis of *Siraitia grosvenorii* revealed different characteristics of green fruit and saccharified yellow fruit. *J Pharm Biomed Anal.* (2017) 145:158–68. 10.1016/j.jpba.2017.06.046 28666162

[15] Cicek SS Esposito T Girreser U. Prediction of the sweetening effect of *Siraitia grosvenorii* (Luo Han Guo) fruits by two-dimensional quantitative NMR. *Food Chem.* (2021) 335:127622. 10.1016/j.foodchem.2020.127622 32739811

[16] Li C Lin LM Sui F Wang ZM Huo HR Dai L Chemistry and pharmacology of *Siraitia grosvenorii*: a review. *Chin J Nat Med.* (2014) 12:89–102. 10.1016/S1875-5364(14)60015-7 24636058

[17] Wang MY Xing SH Luu T Fan M Li XB. The gastrointestinal tract metabolism and pharmacological activities of grosvenorine, a major and characteristic flavonoid in the fruits of *Siraitia grosvenorii*. *Chem Biodivers.* (2015) 12:1652–64. 10.1002/cbdv.201400397 26567944

[18] Xu WK Meng LS. Analysis of sugar content in Siraitiae Fructus. *Guangxi Agric Sci.* (1980) 29.

[19] Ji L. *Effects of Different Drying and Processing Methods on Vitamin C, Color, Phenolics, Antioxidant Activity, and Mogroside V of Luo Han Guo (Siraitia Grosvenorii) Drink.* Master’s tehsis. New Brunswick, NJ: Rutgers, The State University of New Jersey (2016).

[20] Lu FL Li DP Liu JL Huang YL. Chromatographic fingerprinting analysis on chemical compositions of *Siraitia grosvenorii* fruit with different drying treatments. *Guangxi Agric Sci.* (2009) 40:625–8.

[21] Zhou L Zhu YY. The effect of vacuum drying method on the content of ten mogrol glycosides in Siraitiae Fructus by HPLC-MS. *Chin J Pharm Anal.* (2014) 34:275–80. 10.16155/j.0254-1793.2014.02.009

[22] Wang HY Ma XJ Mo CM Zhao H Tu DP Bai LH Determination of sugar components and contents in fruit flesh of *Siraitia grosvenorii*. *Guihaia.* (2015) 35:775–81.

[23] Fichou D Ristivojević P Morlock GE. Proof-of-Principle of rTLC, an open-source software developed for image evaluation and multivariate analysis of planar chromatograms. *Anal Chem.* (2016) 88:12494–501. 10.1021/acs.analchem.6b04017 28193066

[24] Itkin M Davidovich-Rikanati R Cohen S Portnoy V Doron-Faigenboim A Oren E The biosynthetic pathway of the nonsugar, high-intensity sweetener mogroside V from *Siraitia grosvenorii*. *Proc Natl Acad Sci U S A.* (2016) 113:E7619–28. 10.1073/pnas.1604828113 27821754 PMC5127336

[25] Zhang J Dai L Yang J Liu C Men Y Zeng Y Oxidation of cucurbitadienol catalyzed by CYP87D18 in the biosynthesis of mogrosides from *Siraitia grosvenorii*. *Plant Cell Physiol.* (2016) 57:1000–7. 10.1093/pcp/pcw038 26903528

[26] Wang HY Ma XJ Mo CM Zhao H Tu DP Bai LH Effects of shading on contents of mogrosides and sugars in fruit flesh of *Siraitia grosvenorii*. *Guihaia.* (2016) 36:1344–52.

[27] Ames JM. The maillard reaction. In: Hudson BJF editor. *Biochemistry of Food Proteins.* Boston, MA: Springer (1992). p. 99–153.

