# Supplementary material for: Chemical Comparison of Monk Fruit Products Processed by Different Drying Methods Using High-Performance Thin-Layer Chromatography Combined With Chemometric Analysis
Source: Front Nutr. 2022 May 2;9:887992. doi: 10.3389/fnut.2022.887992 (PMC9108421; doi:10.3389/fnut.2022.887992)
Supplement: Supplementary file 1 [file Data_Sheet_1.docx]

Supplementary Material


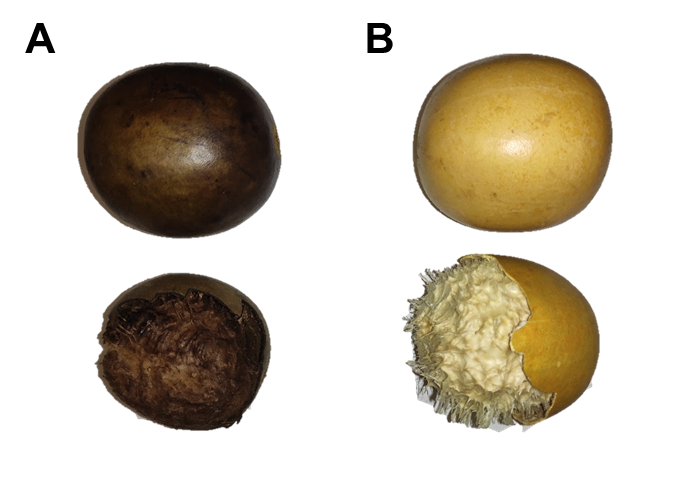


**Supplementary Figure 1.** Appearance of monk fruit dried at (**A**) high temperature and (**B**) low temperature.

**
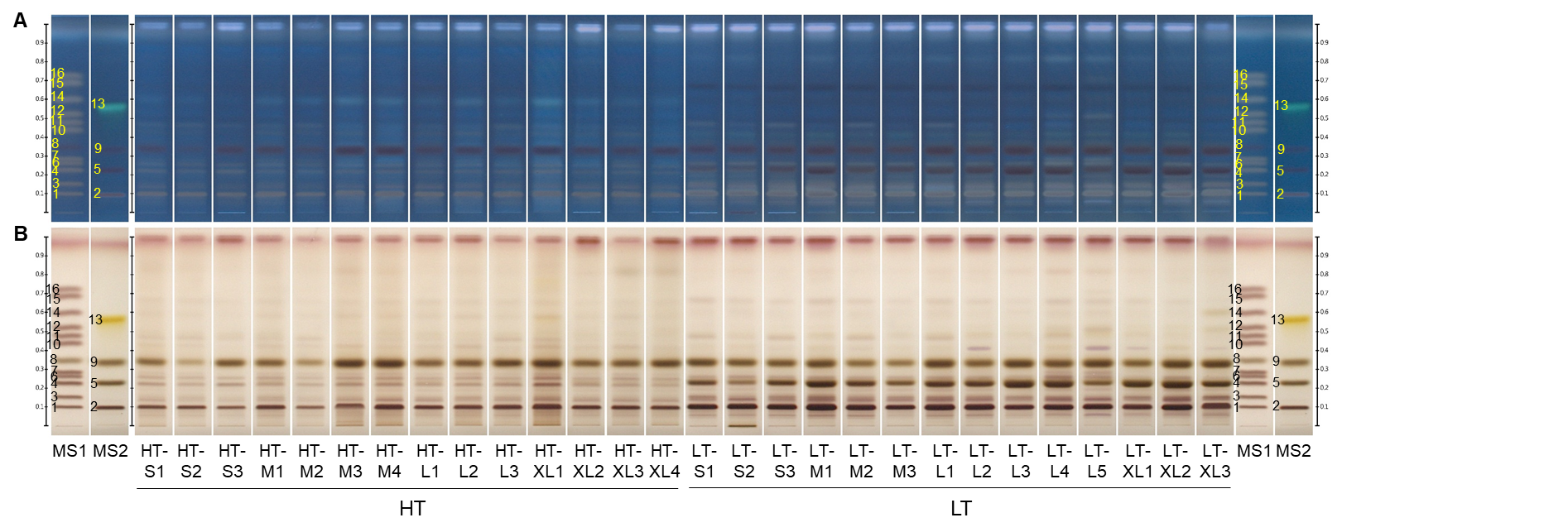
**

**Supplementary Figure 2.** HPTLC chromatograms of mixed standards and monk fruit samples. Plates were immersed into 10% sulfuric acid in ethanol solution and viewed under (**A**) white light and (**B**) UV 366 nm. HT: monk fruit dried at high temperature; LT: monk fruit dried at low temperature; S: small; M: medium; L: large; XL: extra-large; MS1 and MS2: mixed standards. (**1**) mogroside V (R_f_ 0.10), (**2**) 11-oxo-mogroside V (R_f_ 0.10), (**3**) isomogroside V (R_f_ 0.15), (**4**) mogroside IV (R_f_ 0.23), (**5**) sucrose (R_f_ 0.23), (**6**) siamenoside I (R_f_ 0.26), (**7**) mogroside IV A (R_f_ 0.29), (**8**) glucose (R_f_ 0.35), (**9**) fructose (R_f_ 0.34), (**10**) mogroside III A1 (R_f_ 0.44), (**11**) mogroside III E (R_f_ 0.48), (**12**) mogroside III (R_f_ 0.53), (**13**) grosvenorine (R_f_ 0.56), (**14**) mogroside II A2 (R_f_ 0.60), (**15**) mogroside II A1 (R_f_ 0.69), and (**16**) mogroside II E (R_f_ 0.73).

**
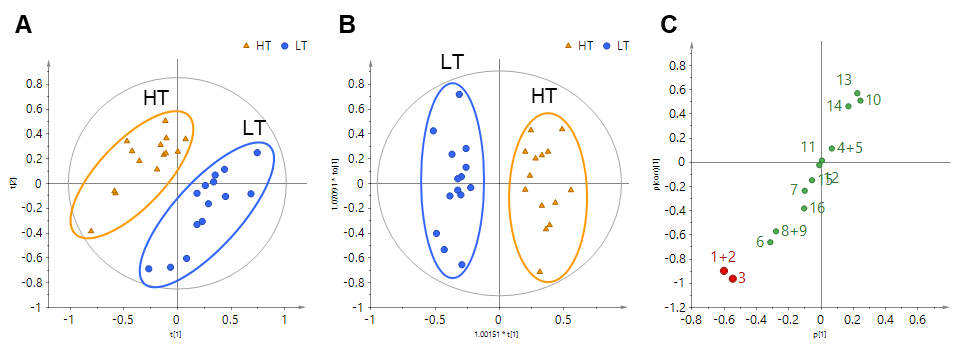
**

**Supplementary** **Figure 3.** (**A**) PCA score plot, (**B**) OPLS‑DA score plot and (**C**) OPLS‑DA S-plot of HT and LT samples based on the data extracted from the 16 metabolites in the HPTLC chromatograms. 1-16 are the same as those in Figure 2. The compounds contributing most to the differences were highlighted with red-filled circles.

**Supplementary Table 1.** **Information of monk fruit samples**

| **No.** | **Code** | **Size** | **Collection date** | **Purchase locations** |
| --- | --- | --- | --- | --- |
| **Samples drying at high temperature** | | | | |
| 1 | HT-S1 | Small | 2020.04.26 | Chengdu, Sichuan, China |
| 2 | HT-S2 | Small | 2020.06.05 | Guangzhou, Guangdong, China |
| 3 | HT-S3 | Small | 2020.12.24 | Guilin, Guangxi, China |
| 4 | HT-M1 | Medium | 2020.03.12 | Chengdu, Sichuan, China |
| 5 | HT-M2 | Medium | 2020.08.20 | Macau, China |
| 6 | HT-M3 | Medium | 2020.10.04 | Zhuhai, Guangdong, China |
| 7 | HT-M4 | Medium | 2020.12.23 | Guilin, Guangxi, China |
| 8 | HT-L1 | Large | 2020.06.05 | Guangzhou, Guangdong, China |
| 9 | HT-L2 | Large | 2020.09.02 | Macau, China |
| 10 | HT-L3 | Large | 2020.10.03 | Macau, China |
| 11 | HT-XL1 | Extra-large | 2020.04.28 | Wenzhou, Zhejiang, China |
| 12 | HT-XL2 | Extra-large | 2020.10.03 | Macau, China |
| 13 | HT-XL3 | Extra-large | 2020.12.20 | Guangzhou, Guangdong, China |
| 14 | HT-XL4 | Extra-large | 2020.12.24 | Guilin, Guangxi, China |
| **Samples drying at low temperature** | | | | |
| 15 | LT-S1 | Small | 2020.09.11 | Guilin, Guangxi, China |
| 16 | LT-S2 | Small | 2020.09.24 | Guilin, Guangxi, China |
| 17 | LT-S3 | Small | 2020.09.26 | Guilin, Guangxi, China |
| 18 | LT-M1 | Medium | 2020.09.11 | Guilin, Guangxi, China |
| 19 | LT-M2 | Medium | 2020.09.17 | Guilin, Guangxi, China |
| 20 | LT-M3 | Medium | 2020.09.24 | Guilin, Guangxi, China |
| 21 | LT-L1 | Large | 2020.09.11 | Guilin, Guangxi, China |
| 22 | LT-L2 | Large | 2020.09.17 | Guilin, Guangxi, China |
| 23 | LT-L3 | Large | 2020.09.26 | Guilin, Guangxi, China |
| 24 | LT-L4 | Large | 2020.12.23 | Guilin, Guangxi, China |
| 25 | LT-L5 | Large | 2020.12.23 | Guilin, Guangxi, China |
| 26 | LT-XL1 | Extra-large | 2020.12.23 | Guilin, Guangxi, China |
| 27 | LT-XL2 | Extra-large | 2020.12.23 | Guilin, Guangxi, China |
| 28 | LT-XL3 | Extra-large | 2020.12.24 | Guilin, Guangxi, China |
